# The Trier Social Stress Test as a paradigm to study how people respond to threat in social interactions

**DOI:** 10.3389/fpsyg.2015.00014

**Published:** 2015-02-02

**Authors:** Johanna U. Frisch, Jan A. Häusser, Andreas Mojzisch

**Affiliations:** Institute of Psychology, University of Hildesheim, HildesheimGermany

**Keywords:** Trier Social Stress Test, social-evaluative threat, cortisol, social support, social cognition, social behavior

## Abstract

In our lives, we face countless situations in which we are observed and evaluated by our social interaction partners. Social-evaluative threat is frequently associated with strong neurophysiological stress reactions, in particular, an increase in cortisol levels. Yet, social variables do not only cause stress, but they can also buffer the neurophysiological stress response. Furthermore, social variables can themselves be affected by the threat or the threat-induced neurophysiological stress response. In order to study this complex interplay of social-evaluative threat, social processes and neurophysiological stress responses, a paradigm is needed that (a) reliably induces high levels of social-evaluative threat and (b) is extremely adaptable to the needs of the researcher. The Trier Social Stress Test (TSST) is a well-established paradigm in biopsychology that induces social-evaluative threat in the laboratory by subjecting participants to a mock job-interview. In this review, we aim at demonstrating the potential of the TSST for studying the complex interplay of social-evaluative threat, social processes and neurophysiological stress responses.

## INTRODUCTION

Stress is a complex interplay of neurophysiological, psychological, behavioral—and also social variables. In their seminal paper, Dickerson and Kemeny (2004) argue that threats to the goal of maintaining the “social self” trigger stress responses, including substantial elevations in cortisol levels. Prototypical situations in which we experience this type of threat are those that bear the danger of a negative evaluation of important and valued aspects of oneself by others (e.g., oral examinations, presentations or job interviews). Such social-evaluative threats have been found to be very potent stressors triggering strong neurophysiological stress responses (see Dickerson and Kemeny, 2004 for a meta-analysis). Responses to these threats include negative self-related cognitions, increases in cortisol, and changes in other neurophysiological variables (Dickerson et al., 2004, 2009). Interestingly, these neurophysiological stress responses are often modulated by social variables (e.g., social support) or their cognitive representations (e.g., the knowledge of belonging to a social group; Häusser et al., 2012). In reverse, social-evaluative threat and the corresponding neurophysiological responses also affect social cognition and social behavior (e.g., Merz et al., 2010; von Dawans et al., 2012).

In order to study both types of relationships in an experimental fashion, a reliable and effective paradigm to induce high levels of social-evaluative threat in the laboratory is needed. The Trier Social Stress Test (TSST; Kirschbaum et al., 1993), which is the gold standard and most commonly employed paradigm in biopsychological stress research (Kudielka et al., 2007), has only recently begun to be used in research examining the interplay between social-evaluative threat, neurophysiological stress responses, social cognition and social behavior. In this review article, we will discuss and integrate the empirical evidence from TSST-studies that have examined research questions related to this interplay.

In what follows, we will first describe the TSST and its variations. We will then summarize and integrate studies that have investigated which social variables (e.g., social support, social status) buffer the neurophysiological stress reaction in response to the TSST. Thereafter, we will turn to studies that have examined the effects of threat-related neurophysiological responses (e.g., cortisol) on social cognition (e.g., social memory) and social behavior (e.g., prosocial behavior). Finally, we will discuss methodological and conceptual issues related to the use of the TSST to study the interplay between social and neurophysiological variables in reaction to threat. Also, we will propose some avenues for future research.

## DESCRIPTION OF THE TSST AND TSST VARIATIONS

**FIGURE 1:**
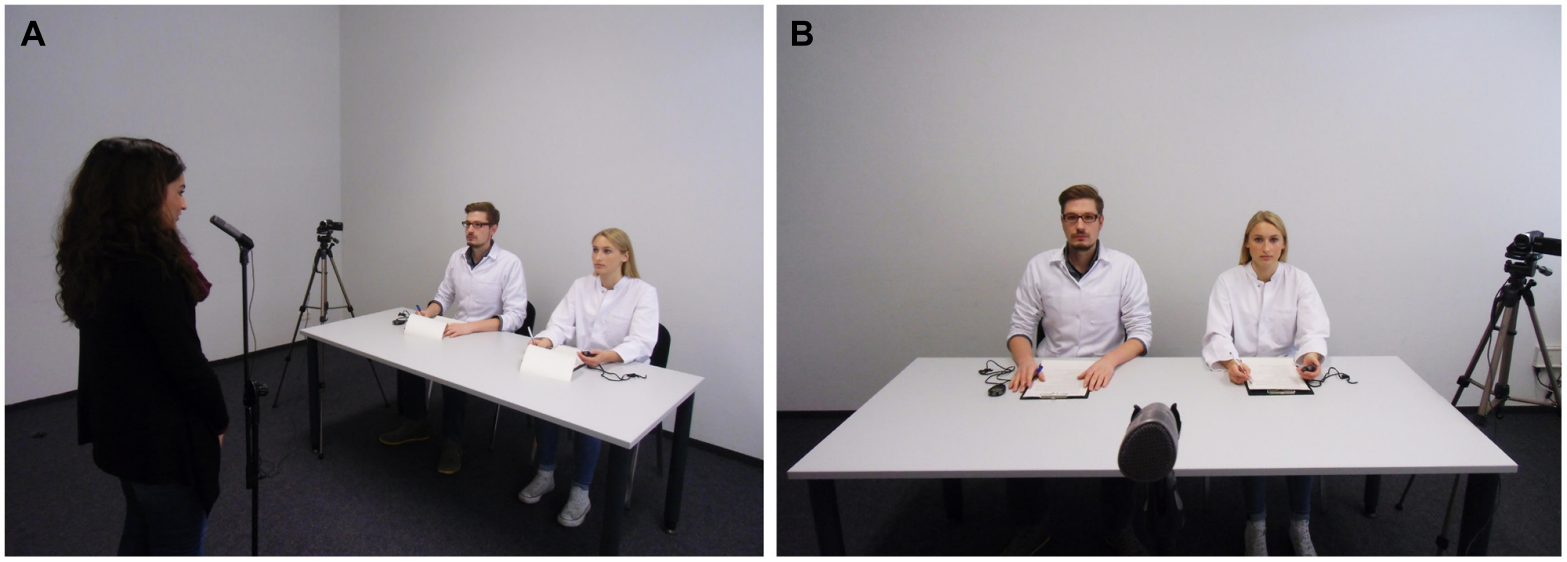
**Set-up of the Trier Social Stress Test (A) and close-up of the two committee members (B)**.

In short, the TSST (Kirschbaum et al., 1993; Kudielka et al., 2007) can be described as a mock job interview. The participants are instructed to imagine having applied for their “dream job” and that they are now invited to a job interview (see **Figure 1**). The TSST consists of three successive phases: (1) A preparation period (3 min), (2) a free speech task in which the participants have to argue why they are the best candidate for the job they wish to apply for (5 min), and (3) a mental arithmetic task in which participants have to sequentially subtract an odd two-digit number from an odd four-digit number (e.g., 17 from 2023; 5 min). The two tasks are performed in front of a selection committee (two or three female and male members), dressed in white lab coats, acting in a reserved manner and providing no facial or verbal feedback. Additionally, participants are video-taped and told that their performance will be evaluated and a voice analysis will be conducted (see Kudielka et al., 2007 for a detailed description of the standard TSST protocol).

The TSST has been found to reliably activate the hypothalamic–pituitary–adrenal (HPA) stress axis and to trigger a two- to threefold release of the stress hormone cortisol (compared to non-stress control conditions) in about 70–80% of participants (Dickerson and Kemeny, 2004; Kudielka et al., 2007). Moreover, various other indicators confirm the stress-inducing potential of the TSST: The activity of the sympathetic–adrenal–medullary (SAM) axis—the other main stress axis besides the HPA axis—can be assessed by changes in cardiovascular parameters (Kirschbaum et al., 1993) or in salivary alpha amylase (Nater and Rohleder, 2009). Additionally the TSST has also been shown to affect immunological parameters (e.g., interleukins, von Känel et al., 2006) and leads to high levels of self-reported stress and anxiety (e.g., Hellhammer and Schubert, 2012). However, since cortisol is the most prominent and most widely assessed indicator of the physiological response in TSST research (Kudielka et al., 2007; Hellhammer et al., 2009), we will focus on cortisol as an indicator of the neurophysiological stress response.

In addition to the standard protocol described above, several variations of the TSST have been developed and validated. The two most important variations concern the development of control conditions for the TSST (Het et al., 2009; Wiemers et al., 2013) as well as the development of a TSST group-version (von Dawans et al., 2011). The Placebo-TSST is a parallelized control condition for the TSST, in which participants have to talk loudly about a movie, novel or holiday trip, and have to do a simple addition task while standing in an upright position, but the social-evaluative component is missing (i.e., no committee, no video camera; Het et al., 2009). For almost all participants, the Placebo-TSST does not lead to a stress response, although it is identical to the TSST in terms of the general procedure, duration, and cognitive and physical load. It is therefore particularly useful when investigating the effects of stress (i.e., as an independent variable) on cognitive or affective outcomes while controlling for cognitive and physical load.

In addition to the Placebo-TSST, another control condition, the friendly TSST (Wiemers et al., 2013), has been developed. Similar to the Placebo-TSST, the participants experience the same cognitive and physical load as participants in the TSST, but additionally they have to perform the tasks in front of a friendly non-threatening committee. To reduce any kind of social-evaluative threat, participants are explicitly told that they are in the control condition, no video cameras are present, the committee members wear no lab coats, and behave in a friendly manner and give positive non-verbal feedback. The friendly TSST has been found to lead to no significant increase in cortisol or increase of negative affect (Wiemers et al., 2013). In its originally proposed version, the friendly TSST does not include a simple addition task like the Placebo-TSST; however, this should be added if also using the TSST in its original form.

The TSST-G (von Dawans et al., 2011) is a group version of the TSST and allows the simultaneous induction of social-evaluative threat in a group of up to six participants. In the TSST-G, the participants stand in a row facing the committee and the video cameras. Participants are separated from each other by dividing walls in order to inhibit social contact between them. The task instructions and sequence are the same as in the original TSST except that participants are asked to perform the tasks one after the other. In the corresponding control condition (Placebo-TSST-G), participants are asked to simultaneously read out a text in a low voice and to simultaneously perform a simple addition task. The TSST-G leads to comparable increases in cortisol levels and self-reported anxiety and stress as the original (individual) TSST. Importantly, in the corresponding Placebo-TSST-G no significant increases in salivary cortisol and self-reported anxiety or stress were observed (von Dawans et al., 2011).

The TSST has also been shown to effectively induce stress and trigger a cortisol response—although somewhat smaller—using virtual reality systems (e.g., Kelly et al., 2007; Jönsson et al., 2010; Merz et al., 2010; Schmid and Schmid Mast, 2013). These variations differ in the presentation of the virtual reality (head-mounted display vs. projections vs. presentation on a TV screen), the used characters (avatars vs. “real” people), the possibility of verbal interaction with the committee (not possible vs. pre-recorded answers) and the number of tasks performed in front of this committee (speech task only vs. speech and mental arithmetic task). These variations bear the advantages of a highly standardized committee-behavior and some of these versions can be used under financial and spatial restrictions (e.g., in an fMRI-scanner).

Moreover, the TSST has also been adapted to different age groups. Buske-Kirschbaum et al. (1997) developed a TSST for children from 7 to 14 years. Instead of the job interview, the children were told the beginning of a story. The children are then asked to finish telling it in front of a committee which—in contrast to the original TSST—provides the children with positive verbal and non-verbal feedback. The mental arithmetic task is also adapted to the numeracy skills of children. When investigating older (retired) adults, the instruction for the job interview can be slightly changed to applying for a part-time job (e.g., child caring, housekeeping; Kudielka et al., 2007). In these two age groups, the TSST also leads to an increase in cortisol (Buske-Kirschbaum et al., 1997; Kudielka et al., 1998). However, for children, the response magnitude seems to be reduced by 30–50% compared to the cortisol response of adults (Buske-Kirschbaum et al., 1997).

## EMPLOYING THE TSST TO INVESTIGATE THE INTERPLAY BETWEEN SOCIAL AND NEUROPHYSIOLOGICAL VARIABLES

Studies that have employed the TSST to examine the interplay between social and neurophysiological variables in reaction to threat can be broadly categorized into two distinct lines of investigation. The first line of investigation deals with the question of which social variables (or the cognitive representation of these social variables) buffer the neurophysiological stress reaction in response to social-evaluative threat (e.g., “Is social support effective in buffering the neuro-endocrine response triggered by the TSST?”). In these studies, the cortisol response to the TSST is the main dependent variable. The second line of investigation focuses on the reverse direction. Here, the TSST is used to induce changes in threat-related neurophysiological responses—mainly increased levels of cortisol—in order to examine their impact on social processes, such as interpersonal behavior or social cognition (e.g., “Do threat-induced elevations of cortisol levels affect social memory or prosocial behavior?”). Hence, in these studies, interpersonal behavior or social cognition are the dependent variables. In what follows, we will review both (a) studies examining the effects of social variables on neurophysiological responses, and (b) studies examining effects of threat-related neurophysiological variables on social processes.

### LITERATURE SEARCH AND STUDY SELECTION

Studies were identified by searching the PsychINFO, MEDLINE, and PSYNDEX databases using the keyword “Trier Social Stress Test” (all text search). This search generated 1003 hits. The abstracts of these hits were checked as to whether the TSST or an adapted version of it was used to investigate a research question related to the interplay between social and neurophysiological variables in healthy adults. Using a snowball search system, reference lists of all identified studies were checked for additional studies that had not been found through the computerized search. The final sample consisted of 17 studies that examined effects of social variables on the neurophysiological stress reaction (Kirschbaum et al., 1995; Hellhammer et al., 1997; Heinrichs et al., 2003; Gruenewald et al., 2006; Ditzen et al., 2007, 2008; Mendes et al., 2007; Taylor et al., 2007, 2010; Cosley et al., 2010; Page-Gould et al., 2010; Chen et al., 2011; Buchanan et al., 2012; Häusser et al., 2012; Schmid and Schmid Mast, 2013; Engert et al., 2014; Frisch et al., 2014) and nine studies that examined effects of threat-related neurophysiological reactions on social cognition and social behavior (Takahashi et al., 2004; Roelofs et al., 2005; Smeets et al., 2009; Merz et al., 2010; Starcke et al., 2011; von Dawans et al., 2012; Leder et al., 2013; Vinkers et al., 2013; Tomova et al., 2014).

### EFFECTS OF SOCIAL VARIABLES ON NEUROPHYSIOLOGICAL RESPONSES

In this part, we will review studies that address the question of how social processes (e.g., social support) or the cognitive representation of social processes (e.g., social status) affect the neurophysiological response (e.g., the release of cortisol) to social-evaluative threat.

#### Social support

In our lives, we face countless situations in which we are observed and evaluated by other people and our social self is threatened (e.g., oral examinations or job interviews). Fortunately, in some of these situations we are not alone but receive support from others. There has been a wealth of research on the effectiveness of social support in stressful situations (see Uchino et al., 1996; Thorsteinsson and James, 1999 for a meta-analysis and a review). Intriguingly, although social support is often effective, there are also situations in which social support has no (Taylor et al., 2010) or even detrimental effects (e.g., Maisel and Gable, 2009). Experimental research in the laboratory using the TSST as a paradigm to induce social-evaluative threat has helped to identify some important qualifications of the effect of social support.

At first glance, it may seem counterintuitive to invite participants to the laboratory in order to study the effects of social support—something that we experience regularly in our lives and that might, hence, be studied best in those real life contexts (e.g., via questionnaires or diary studies). However, an experimental approach to study the effects of social support offers at least two major advantages: First, using an experimental approach, and especially the TSST, guarantees that all participants are confronted with the very same kind of stressor. Second, within the TSST protocol the properties of the support situation can be exactly determined by the experimental manipulation of (a) the characteristics of support recipient and provider (e.g., sex, personality), (b) the relationship between support recipient and provider (e.g., sharing of a social identity), (c) the type of support provided (e.g., verbally or non-verbally; emotional or instrumental) or (d) the availability of other hormones (i.e., oxytocin) in order to target the underlying mechanism of the beneficial effect of social support on cortisol. In what follows, we will discuss and integrate the findings from studies that have addressed these moderators of social support.

***Characteristics of support recipient and provider.*** One of the first TSST studies investigating the stress-buffering effects of social support was conducted by Kirschbaum et al. (1995). In their study, male and female participants either received social support by their romantic partners, by an opposite-sex stranger (i.e., a trained confederate) or no support immediately before the TSST. The support was provided verbally and entailed aspects of emotional and instrumental social support (e.g., focusing on positive appraisals and information on effective self-presentation). Surprisingly, social support attenuated the stress reaction only for men. Thus, men showed a lower cortisol response when supported by their partners and a tendency for attenuation when supported by strangers. Women, however, did not benefit from social support; they even showed a tendency for increased levels of cortisol when obtaining support from their partners. This pattern was not found for the psychological stress response: In all conditions participants reported a moderate level of stress. Furthermore, self-reported stress was not associated with the cortisol response. At first glance, it seems that the effectiveness of social support is moderated by the sex of the support receiver and by familiarity with the support provider. But importantly, since the sex of the support provider was not included as an experimental factor, the sex of the support provider and recipient were confounded (i.e., male participants were always supported by females and vice versa). Therefore it remains unclear if the results are due to gender differences in support reception or support provision (Kirschbaum et al., 1995). The stress-buffering effect of social support was confirmed by a study by Ditzen et al. (2008). Specifically, Ditzen et al. (2008) suggested that the attachment style of the support recipient might also play a crucial role in the effectiveness of social support. In their study, male participants either received verbal social support by their romantic partners prior to the TSST or received no support. Additionally, the attachment style of the participants was assessed via self-reports. Attachment styles can be described on the two dimensions attachment anxiety (i.e., fear of losing the partner) and attachment avoidance (i.e., striving for independence from the partner; Fraley et al., 2000). Ditzen et al. (2008) found that social support buffered the cortisol response independent of attachment style—particularly during the phase of threat anticipation. In contrast to Kirschbaum et al. (1995), a stress-buffering effect of social support was found for psychological stress, too. More specifically, this effect was moderated by attachment style, that is, social support buffered psychological stress only for securely attached men (i.e., low on the anxiety and low on the avoidance dimension). In contrast, for insecurely attached participants, social support had no effect (compared to the no support condition).

Apart from the sex of the recipient (or provider) of social support, a further moderating variable for the effectiveness of social support may be the cultural background of the support recipient and the specific type of social support (i.e., explicit vs. implicit support). Taylor et al. (2007) proposed that for Asian Americans explicit support (i.e., seeking and using emotional and instrumental support) should have detrimental effects, because they are afraid of potential negative consequences that this focus on their own needs might have for their relationship with others. Instead, they should benefit more from implicit types of social support (i.e., reminiscence of belonging to a valued social group). In order to test this hypothesis, immediately before the start of the TSST, Asian Americans and European Americans were asked to either write a letter to a friend in which they asked for his or her support in the upcoming task (explicit support), to think and write about a close group (implicit support) or to work on an unrelated task (no support). As predicted, for Asian Americans explicit support led to a higher cortisol response and to more self-reported stress as compared to the implicit support and no support condition. Conversely, European Americans showed a higher cortisol response when faced with implicit support as compared to the explicit and no support condition, but there was no difference in self-reported stress between the groups.

It is important to note that in the studies described above social support was always provided by a third person (i.e., a confederate, friend, or partner) that was not directly involved in the stress situation. However, in many real life situations one cannot rely on one’s partner or best friend providing support (you would definitely not bring them along to a job interview). As is often the case, the only potential source of social support available is the stressor him- or herself: Imagine, for example, an oral examination in school or at university. Wouldn’t it be nice if you had a teacher to emotionally support you? Surprisingly, the results of a study by Taylor et al. (2010) suggest that it would not really matter; social support offered by the source of threat (i.e., the two members of the TSST committee) was not effective. In their study, Taylor et al. (2010) manipulated the behavior of the TSST-committee. Instead of being neutral and providing no feedback (as in the standard version of the TSST), the committee behaved either non-verbally supportive (e.g., by leaning forward and smiling) or non-verbally unsupportive (e.g., by showing signs of boredom like frowning or sighing). Strikingly, compared to a control condition without any committee, participants in both committee-present-conditions showed an equally strong cortisol response. In other words, a supportive TSST committee did not attenuate the cortisol stress reaction. Cosley et al. (2010) suggested that whether or not support by the stressor is supportive is moderated by inter-individual differences in the trait “compassion for others.” People with high levels of compassion should perceive the support provider as being more compassionate, interpreting the offered support in a more positive way. Similar to Cosley et al. (2010), Taylor et al. (2010) manipulated the behavior of the TSST-committee. Half of the participants faced an emotionally supportive committee whereas the other half faced the neutral standard TSST-committee. Consistent with their expectations, in the social support condition participants with high levels of compassion for others showed lower cortisol responses than those with low levels of compassion. However, in the neutral committee behavior condition, compassion was not associated with the cortisol response.

Taken together, the five studies reviewed above have identified important moderators of the effect of social support on the neurophysiological response to social-evaluative threat, such as sex (Kirschbaum et al., 1995), attachment style (Ditzen et al., 2008), cultural background (Taylor et al., 2007) and personality (Cosley et al., 2010) of the support recipient. Furthermore, characteristics of the support provider also influence the effectiveness of social support: Support by the stressor itself is not per se effective (Taylor et al., 2010) and, at least for men, support from their romantic partner tended to be more effective than support by a stranger (Kirschbaum et al., 1995). This last finding suggests that the relationship between provider and recipient of social support might be an additional important moderator that might also play a role in explaining the finding of Taylor et al. (2010).

***Relationship between provider and recipient.*** In line with this idea, Frisch et al. (2014) proposed that the results of Taylor et al. (2010) can be explained when taking the relationship between support recipient and provider into account. Building on the social identity approach (Haslam, 2004), Frisch et al. (2014) extended Taylor et al.’s (2010) original study design by including a manipulation of social versus personal identity as an additional experimental factor. In order to do so, the TSST committee consisted of two confederates who pretended to be real participants and who were designated as the TSST committee by a faked drawing of lots procedure. Prior to the TSST, the salience of either a shared social identity (i.e., a feeling of “we”-ness) between the participant and the TSST committee or a personal identity was manipulated: To make a social (vs. personal) identity salient, participants had to wear same (vs. different) colored T-shirts, were asked to think of similarities (vs. differences) and worked alone on an idea generation task in which group (vs. individual) performance was analyzed. In the following TSST, similar to Taylor et al. (2010), half of the participants faced an emotionally supportive committee whereas the other half was confronted with an unsupportive committee. As hypothesized, a stress-buffering effect of social support (i.e., a decreased cortisol reaction) was only found in the social identity condition. In the personal identity condition, the results resembled the findings of Taylor et al. (2010), that is, participants showed the same cortisol reaction regardless of being supported or not. Interestingly, for self-reported stress, no stress-buffering effect of social support in the social identity condition was found. Taken together, the study by Frisch et al. (2014) suggests that social support buffers the cortisol stress reaction only if a shared social identity between the provider and recipient of support has been established.

Whereas Frisch et al. (2014) showed that a shared social identity is an important moderator of the effectiveness of social support, Häusser et al. (2012) found that a shared social identity per se can be an effective stress-buffer. In their study, participants underwent either the TSST-G (von Dawans et al., 2011) or the Placebo-TSST-G in groups of four. Beforehand, for half of the participants a shared social identity with their fellow group members was made salient and for the other half a personal identity was activated. As expected, a shared social identity worked as a stress-buffer. Thus, participants in the shared social identity condition showed a significantly reduced cortisol reaction in response to the TSST-G. Importantly, since participants were not allowed to interact with each other during the whole study, no overt transmission of support was possible. In other words, the mere cognitive representation of belonging to the same social group (i.e., “we are going through this together”), buffered the cortisol reaction in response to social-evaluative threat. Again, this stress-buffering effect was not found for self-reported stress. This study provides a nice example of how even a cognitive representation of social processes can be effective in coping with social-evaluative threat.

In sum, especially the study by Frisch et al. (2014) highlights that when facing social-evaluative threat a shared social identity is an important precondition to benefit from support. A shared social identity may provide group members with a common interpretive framework (things are perceived and evaluated in a similar fashion by group members) and may increase feelings of trust. These processes may facilitate the interpretation of the offered social support as wholehearted and in the spirit it was intended thereby making the provision of support more effective (Haslam et al., 2012; van Dick and Haslam, 2012).

However, two limitations of this research have to be put forward: First, in both studies the predicted stress buffering effects were only found for the neuroendocrine stress reaction but not for self-reported stress (see also Kirschbaum et al., 1995; Ditzen et al., 2007 for similar findings). Second, the specific mechanisms mediating the effects of social identification on the neuroendocrine stress reaction are still far from clear.

***Type of support.*** It is not only the characteristics of support recipient and provider and their relationship that should be taken into account, but also the type of support. For example, as already described above, in the study by Taylor et al. (2007), the effects of the cultural background of the recipients of social support were moderated by the type of support (explicit vs. implicit support). In another study by Ditzen et al. (2007), female participants received either (a) verbal support or (b) a shoulder massage from their romantic partner or (c) received no support prior to the TSST. In line with Kirschbaum et al. (1995), women who received verbal partner support did not profit from it and showed a similar increase in cortisol as compared to the no support control condition. However, since verbal support was provided exclusively from male partners it remains unclear whether this finding results from an ineffective support provision by males, or ineffective support reception by females. In contrast, women who received a massage had an attenuated cortisol reaction. For self-reported stress and anxiety, no differences between the three conditions were found. Hence, although a stress-buffering effect on the physiological stress response was found, again, this was not found on the subjective-psychological level.

***Availability of oxytocin.*** Recently, the activity of the hormone oxytocin has been discussed as one underlying biological mechanism of the stress-buffering effect of social support (see Campbell, 2010; Hostinar et al., 2014 for reviews). Predominantly in studies with animals, but also in some human studies, it has been shown that oxytocin is released in positive social contexts and that it has dampening effects on the activity of the HPA axis (Hostinar et al., 2014). However the direct mediation of the stress-buffering effect of social support in situations of social-evaluative threat has not been demonstrated so far, which may be partly due to problems with measuring peripheral oxytocin (McCullough et al., 2013).

Heinrichs et al. (2003) investigated the stress-buffering effects of both verbal support and oxytocin for male participants. Specifically, in addition to the manipulation of social support (support by best friend vs. no support), they also administered intranasal oxytocin to half of the participants whereas the other half received a placebo about an hour prior to the TSST. The results showed that social support as well as oxytocin suppressed cortisol responses, with participants receiving both treatments having the lowest cortisol response. These findings suggest that oxytocin is involved in the down-regulation of the HPA-axis of humans and that it enhances the beneficial effect of social support. However, the study design employed by Heinrichs et al. (2003) does not allow testing of whether the effect of social support was mediated by oxytocin secretion (i.e., social support increases oxytocin levels which, in turn, buffer the cortisol reaction). More evidence comes from a study of Chen et al. (2011). They investigated whether variations in the receptor gene of oxytocin are associated with the stress-buffering effect of social support. One special single-nucleotide polymorphism in this gene (rs53576) has been found to be related to reduced social abilities (e.g., Bakermans-Kranenburg and van Ijzendoorn, 2008; Tost et al., 2010) and less searching for social contact (Kim et al., 2010). Chen et al. (2011) hypothesized that participants carrying this special allele variation might also profit less from social support. In their study, male participants either received social support from a female friend or no support prior to the TSST-G. As predicted, rs53576 G carriers seemed to benefit more from social support than individuals with the AA genotype, who showed almost identical subjective and cortisol stress reactions in both the support and no-support conditions. These results indicate that genetic variations of the oxytocin system modulate the effectiveness of social support as a buffer against social-evaluative threat. Again, however, these results provide no direct evidence for the idea that the stress-buffering effects of social support are mediated by oxytocin. Rather, the findings point to an interactive effect of social support and oxytocin. Since oxytocin has been found to increase trust (Kosfeld et al., 2005), it is tempting to speculate that the interactive effects of oxytocin and social support are due to the fact that oxytocin increases the probability that the recipient of support trusts more in the wholeheartedness of the provided support, thereby making the provision of support more effective. Future research is needed to test this hypothesis.

### Observation of threat

In almost all of the studies employing the TSST to examine how social variables influence neurophysiological responses, the focus is on the individual being threatened and his or her reaction to the TSST. By contrast, two recent studies shifted this focus toward the neurophysiological reactions of the persons being the stressor (i.e., the committee members; Buchanan et al., 2012) or to persons who observed the TSST participant (Engert et al., 2014). Particularly, both studies investigated the relationship between the neurophysiological responses of the stressor/observer and the neurophysiological responses of the participants being exposed to the TSST. In other words, these studies aimed at exploring whether the response of the stressor/observer resonates with that of the participant. Physiological resonance means that the stress response of the stressor/observer is a function of the stress response of the TSST participant (Engert et al., 2014). In the study of Buchanan et al. (2012), the cortisol responses of the TSST-participants indeed were predictive for the cortisol response of the TSST-committee members (i.e., trained research assistants). Moreover, cortisol responses were generally higher for those committee members with higher levels of trait empathy, indicating that empathy plays a crucial role in physiological resonance. However, the direction of the effect is not entirely clear. It is easy to imagine that the TSST can also be a stressful experience for the committee members (Engert et al., 2014). For example, being responsible for the distress of another person, or the demand to suppress spontaneous supportive behavior (such as smiling or nodding) could lead to stress in the TSST-committee. Therefore, it seems also possible that a stressed committee causes more stress in the participant accounting for the relationship between the cortisol responses. In order to investigate if a real empathic stress response can be elicited by the TSST, Engert et al. (2014) used a different approach. A passive observer (either a stranger or a romantic partner) witnessed the participant undergoing the TSST either through a one-way mirror or via video. Hence, in contrast to Buchanan et al. (2012), the participant could not see the observer and could therefore not be influenced by his or her stress response. Twenty-six percent of all observers showed a significant increase in cortisol levels, with the strongest cortisol responses found in observers watching their own partners through a one-way mirror. Furthermore, Engert et al. (2014) also demonstrated resonance since the cortisol stress response of the observers was to some degree predicted by the cortisol stress response of the participants. The finding that the neuroendocrine responses of the observer/stressor and the participant resonate is intriguing. Although emotional contagion—the “catching” up of the emotion of the interaction partner (Hatfield et al., 1993)—has been shown at a behavioral level (e.g., facial mimicry, Mojzisch et al., 2006), or at a cardiovascular level (Konvalinka et al., 2011), these two studies are the first to show this resonance of stress on a neuroendocrine level. However, one limitation of both studies is that they do not address the underlying mechanism of this effect. Both studies highlight the importance of empathy, but it remains completely unclear what cues (e.g., facial expression, voice, and other behaviors) of the threatened participants trigger the HPA axis activity in the observer. Furthermore, since no self-reports of the stressor/observers were obtained we do not know which feelings accompany this HPA activation. As Dickerson et al. (2004) suggested and have shown empirically (Gruenewald et al., 2004; Dickerson et al., 2008), the threat of one’s social self is associated with self-conscious emotions and cognitions (i.e., shame or embarrassment). Hence, it would be interesting to examine whether the same feelings are triggered in the observer.

Furthermore, at this point, we can only speculate about the implications of this resonance, but it might enhance the understanding for the situation or for the needs of the threatened person. Moreover, it might enhance the provision of social support or might also have beneficial effects for the long-term relationship between the threatened person and the observer. Future research should address these implications as well as the psycho-physiological pathways of transmission of this resonance.

### Social status and power

Social self-preservation theory (Dickerson et al., 2004, 2009) argues that social-evaluative threats trigger a coordinated psychophysiological and behavioral response in order to prevent negative effects, like loss of status or social exclusion. Gruenewald et al. (2006) hypothesized that this relationship is moderated by the social status of the individual. Individuals with low status should react stronger to additional threats of their already low status, as compared to high status individuals. To test this hypothesis, Gruenewald et al. (2006) assessed the subjective self-reported social status from college students living in a residential dormitory before letting them undergo either the TSST or the Placebo-TSST. In stark contrast to their expectations, they found that only students high in social status exhibited the typical cortisol reaction whereas students low in status showed a blunted cortisol reaction. These findings are, however, in line with a study by Hellhammer et al. (1997) who observed male army recruits during boot camp training over several weeks and confronted them with the TSST. The recruits were divided into small groups, and the social status of each of the recruits was assessed somewhat more objectively than in the study of Gruenewald et al. (2006) by asking every recruit to indicate how he perceived his fellow recruits. In reaction to the TSST, the recruits with a higher status showed the strongest cortisol reaction whereas low status recruits only showed a weak response.

Albeit speculatively, these findings could be explained by lower ego-involvement of participants low in status. Low status participants might be in general less concerned with evaluation situations—which could also be seen as one reason for their low status (Hellhammer et al., 1997; Gruenewald et al., 2006). In contrast, for individuals with a high status there might have been more at stake and therefore their fear of a potential status loss led to an increase in cortisol (Hellhammer et al., 1997).

Note that these two studies (Hellhammer et al., 1997; Gruenewald et al., 2006) investigated the influence of the status of individuals in already existing groups; hence a quasi-experimental design was used. Since quasi-experimental designs are prone to the influence of confounding variables, they should be supplemented with more controlled experimental studies. Such a study was conducted by Schmid and Schmid Mast (2013) who actually found the exact opposite pattern of results as the two previous studies (i.e., their results were in support of the original proposition of Gruenewald et al., 2006). In two experiments, using adapted TSST versions, the experimental priming of high power in social situations compared to low power resulted in a weaker increase in heart rate (unfortunately cortisol was not assessed). Moreover, participants in the high power condition reported less fear of evaluation, showed less non-verbal signs of nervousness and performed better in the speech task, which was rated by different raters based on the recorded videos.

In sum, these three studies convey an inconclusive picture on the effects of social status on the reaction to threat. Although all three studies confirmed that experience of previous social interactions (e.g., the emergence of status in a social group) affects the stress response and even the performance, the direction of this effect remains unclear for the present. The picture might be even more complex when acknowledging that power and status can be thought of as slightly different concepts, for example, a relatively high status does not necessarily go along with high levels of power in social situations (e.g., the recruits high in status in the study of Hellhammer et al., 1997 were also dependent on their supervisors). Further research is therefore needed to specify the conditions under which high status/power has a protective effect.

Interestingly, much of the current research on the influence of rank on reactions to stress is based on animals, but here results are often inconsistent (see Sapolsky, 2005 for a review). Sapolsky (2005) suggests that there are several moderators (i.e., stability or personality) that determine whether primates of low or high status experience more stress: For example, when hierarchies are rather stable then individuals with low ranks experience more stress than those with high ranks, whereas in the case of unstable hierarchies the relation is inversed. In the context of inter-group competition, a corresponding pattern has been found for humans (Scheepers and Ellemers, 2005; Scheepers, 2009). Scheepers (2009) found that when hierarchies were stable, members of low status groups, if confronted with an inter-group competition, exhibited a more pronounced cardiovascular threat pattern (e.g., Blascovich and Mendes, 2000) than members of high status groups. By contrast, when hierarchies were unstable, members of low status groups showed a challenge pattern, whereas members of high status groups displayed a threat pattern. Since the underlying motivation of maintaining a positive (social) self is very similar in situations of inter-group threat and in those of social-evaluative threat, stability of hierarchies might also be an important moderator in these latter situations.

### Racial bias/intergroup threat

A mounting body of evidence shows that interacting with people of different races can produce threat and stress reactions (e.g., Mendes et al., 2002). However, the neuroendocrine reactions to intergroup threat are likely to be shaped by the individuals’ racial bias. To test this idea, Mendes et al. (2007) examined the influence of implicit racial attitudes of White participants on their neurophysiological reaction toward a TSST in which the committee members were either part of an in-group (i.e., White) or of an out-group (i.e., Black). The implicit racial attitudes were assessed with the Implicit Association Test (Nosek et al., 2005). The results revealed that the cortisol response did not depend on the group membership of the committee members or the racial-biases of the participants. However, more egalitarian attitudes of the participants were associated with a more salutary stress response (as defined by the ratio of the hormone dehydroepiandrosterone to cortisol): For participants facing Black committee members, a low racial bias was associated with a more salutary stress response, a lower report of threat appraisals, and less signs of anxiety than a high racial bias. Page-Gould et al. (2010) replicated and extended this study by including both White and Black participants. Thus, Black and White participants underwent the TSST facing either a Black or White committee. The results of this study confirmed that the cortisol reaction in response to the TSST did not depend on whether the committee members were part of the in-group or the out-group. However, the amount of prior intergroup contact—which was assessed beforehand—was positively related to the physiological recovery in both intergroup conditions (i.e., Black participants and White committee members or White participants and Black committee members). Thus, participants reporting more prior intergroup contact had a steeper decline in cortisol levels following an intergroup stressor than participants with only few prior intergroup contacts. Interestingly, the race of the participants did not moderate these results.

Taken together, these two studies highlight that not only specific behaviors of the interaction partner in situations of social-evaluative threat can affect neurophysiological responses, but also stereotypes and intergroup contact. Both studies, however, did not find effects on the immediate cortisol reaction, but rather on indicators of recovery. This is an important finding suggesting that social variables may not only exert influences on the immediate cortisol reaction but also on the rate of recovery from stressful events. Since especially the failure to recover from such events may have several negative health consequences (e.g., McEwen, 1998), it seems worthwhile to focus not only on peak neuroendocrine responses but also to analyze rates of recovery (see Linden et al., 1997 for a review).

## EFFECTS OF NEUROPHYSIOLOGICAL RESPONSES TO THREAT ON SOCIAL PROCESSES

All of the studies reviewed so far used the TSST as a tool to test whether the manipulation of specific social variables (e.g., social support) buffers the neurophysiological stress response. In these studies, the cortisol response to the TSST is the main dependent variable. By contrast, a different line of research examines the social-cognitive effects of stress. In these studies, stress is the independent variable (i.e., TSST vs. Placebo-TSST) and the main dependent variables are participants’ social cognitions or behaviors in response to this manipulation. For example, this line of research has tested whether acute stress affects prosocial behavior. In the next section, we will focus on this second line of research.

### Social cognition/social memory

Social cognition, defined as “the mental operations that underlie social interactions and includes the ability to attribute mental states (e.g., emotions, thoughts, intentions) to oneself and others” (Smeets et al., 2009, 507), is one essential prerequisite for successful interactions. Smeets et al. (2009) investigated the influence of social-evaluative threat on the ability to infer the non-emotional and emotional states of other individuals. After the TSST/Placebo-TSST, participants were asked to indicate the emotional and non-emotional states of different characters in a short movie. The results showed an effect of threat on the ability to infer the states of other individuals which, however, was moderated by sex as well as by the magnitude of the cortisol response: When exposed to social-evaluative threat, male high-cortisol responders were better at identifying emotional, and non-emotional states than male low-cortisol responders, however, they were not better than the non-stressed control group. By contrast, female participants showed the opposite pattern, that is, when exposed to social-evaluative threat, female low-cortisol responders performed better than female high-cortisol responders and the non-stressed control group. However, these results are somewhat inconsistent with the results of a later study by Tomova et al. (2014). In this study, the ability to distinguish between the self and the other—another import prerequisite for empathy and mentalizing—was assessed in three tasks (e.g., a perspective taking task in which participants had to arrange objects according to instructions of another person with a different visual perspective). For women, the ability to distinguish between the self and the other was increased under conditions of social-evaluative threat (TSST-G vs. Placebo-TSST-G), whereas it was decreased for men. Moreover, in this study, cortisol was not correlated with the ability of self-other-distinction.

From a more theoretical point of view, it is plausible that abilities such as emotion recognition should be enhanced during threat—particularly in women: The tend-and-befriend model of Taylor et al. (2000) posits that women do not respond to stress with fight-or-flight (cf. Cannon, 1932) as men do, but show a more affiliative stress response. This involves nurturing behavior in order to protect the offspring (tending) as well as activities to create and maintain the social network (befriending). Improved emotion recognition and increased empathy are beneficial in forming these social bonds (Tomova et al., 2014). Future research is needed to confirm and to disentangle the diverging finding of these two studies.

In another study, Leder et al. (2013) investigated the effects of social-evaluative threat on the ability of strategizing in a decision making context. The ability of strategizing—that is, thinking about what other actors might think and do—is important for many decision situations, especially in economic decision-making where asset prices are less affected by the fundamental value of the asset but more by what people think everyone else thinks the asset value is. This ability was assessed by using the Beauty Contest game: Following previous research (e.g., Nagel, 1995), four participants were asked to choose a number between 0 and 100. They were told that the participant whose number is closest to the average of all chosen numbers multiplied by 2/3 will be the winner of this game. Hence, in order to win this game, participants have to anticipate the answers of the other participants. For example, participants who show no signs of strategic reasoning would pick a random number. However, participants with a higher level of reasoning would pick numbers around 33, since they assume the other participants would have chosen random numbers (which would result in a mean around 50 that then has to be to be multiplied by 2/3). Furthermore, if a participant expects that all other participants will figure this out, then he or she would choose a number close to 22.22. With increasing iterations, the number converges toward zero (i.e., the Nash equilibrium). Leder et al. (2013) found that threatened male participants (TSST-G) chose higher numbers in the beauty contest game than non-threatened individuals (Placebo-TSST-G), indicating lower levels of strategic reasoning. The relationship between social-evaluative threat and strategic decision making was mediated by the threat-induced increase in cortisol. Additional analyses revealed that it took stressed individuals longer to learn and understand the strategic nature of the game compared to participants in the control group. This is in line with previous findings on the effects of stress on impaired feedback processing (Starcke and Brand, 2012), but might also be due to impaired mentalizing, that is, the ability to anticipate what interaction partners might think or do (Tomova et al., 2014).

Apart from having the ability to tune into others or to anticipate their behavior, it is important for social interactions to encode, memorize and update important information about the interaction partner. Stress and cortisol have been found to negatively affect the retrieval of declarative memory contents (see Wolf, 2009 for a review). Two studies show that this also holds true for the memory retrieval of social information. For example, Takahashi et al. (2004) found an impaired social memory for face-name associations under conditions of social-evaluative threat in men, compared to a non-stressed participant. Moreover, cortisol was negatively associated with performance in the memory task. However, since both the encoding and retrieval took place after the TSST, it remains unclear which process was affected by threat. To disentangle the potential effects on encoding and retrieval, Merz et al. (2010) conducted a study in which male and female participants had to learn biographical information about two persons (e.g., gender, hometown, birth date) before the TSST and were asked to recall them after being exposed to the TSST. Compared to their own performance in a control session without social-evaluative threat, participants made more mistakes in the retrieval of the biographical material after being threatened. Furthermore, and in line with Takahashi et al. (2004), cortisol levels were negatively associated with recall performance. Interestingly, social-evaluative threat and cortisol have been found to also affect declarative memory—for non-social contents—positively (e.g., Kuhlmann and Wolf, 2006). Whether memory is positively or negatively affected seems to be dependent on the memory processes involved; whereas cortisol has a negative influence on memory retrieval, it has a positive effect on memory consolidation (Wolf, 2009). Future research should aim at examining whether these positive effects of threat and cortisol can also be found for the consolidation of social memory contents.

In sum, these findings suggest that social-evaluative threat has mostly negative effects on emotion recognition in others, self-other distinction, strategizing, and memory retrieval for social information. These findings are remarkable insofar as emotion recognition or effective updating of social information are valuable resources when coping with social-evaluative threat. Hence, social-evaluative threat negatively affects the very abilities that are needed to cope with threat. In other words, an ancient biological stress response interferes with the social-psychological demands of typical modern threats.

### Approach/avoidance behavior

Roelofs et al. (2005) sought to investigate how social-evaluative threat influences approach and avoidance behavior to social stimuli. To this end, they used a computerized approach-avoidance task, in which participants saw either happy or angry faces and either had to push a button requiring arm flexion (i.e., approach behavior) or had to push a button requiring arm extension (i.e., avoidance behavior) in response to these stimuli. Employing a within-subjects-design, male and female participants were tested both before and after the TSST. Before the TSST, participants showed the well-established congruency effect—a faster reaction in trials in which movement and stimulus were congruent (i.e., angry face and arm extension, happy face and arm flexion) than in incongruent trials (Solarz, 1960). However, after the TSST, participants who had a high cortisol response no longer showed the congruency effect, that is, the reaction times in the congruent trials became slower and similar to those in the incongruent trials. This effect was not evident in low cortisol responders. Albeit speculatively, increased reaction times can be considered as a freezing reaction, similar to what has been found in animal studies, where neither a preference for avoiding nor approach behavior exists anymore (Roelofs et al., 2005). Hence, similar to the effects on social cognition and social memory retrieval, stress has a dysfunctional effect on behavior actually needed to effectively cope with the stressful situation. Although the classic perception of the human stress reaction is that it is functional in helping the organism to overcome the stressful event (e.g., by supplying it with additional energy), this may not necessarily be the case when it comes to social-interactive coping resources.

### Prosocial behavior

Given that social support and social identification in groups are likely to buffer the neuroendocrine stress reaction in response to social-evaluative threat (e.g., Häusser et al., 2012), engaging in prosocial behavior (e.g., providing help and support to each other) might be a functional response to social-evaluative threat. Building on this notion, von Dawans et al. (2012) examined the effect of social-evaluative threat on subsequent prosocial and antisocial behavior. They found that after being exposed to the TSST-G, male participants were more prosocial in economic games. Compared to the non-stressed control group (Placebo-TSST-G), they trusted their partners more, were themselves perceived more trustworthy and shared more money with others. Importantly, the results also suggest that prosocial behavior following social-evaluative threat is not due to an unspecific increase in the readiness to bear risks. Thus, social-evaluative threat specifically affected the willingness to accept risks arising through social interactions, whereas non-social risk taking was not affected. Also, the stress manipulation had no influence on negative social interactions (i.e., punishment behavior). Taken together, this study provides first experimental evidence suggesting that men—and not only women—engage in prosocial tending-and-befriending behavior in response to stress (Taylor et al., 2000).

Similarly, Vinkers et al. (2013) investigated the effects of social-evaluative threat on reactions to unfair offers in an ultimatum game. In an ultimatum game, one player is given a sum of money that he or she can allocate between herself and another player. The recipient has the option of accepting or rejecting this offer. If the offer is accepted, the sum is divided as proposed. If it is rejected, neither player receives anything. In addition, Vinkers et al. (2013) used a one-shot variant of the Dictator Game in which participants received 10€ with the possibility to donate any amount to “Unicef” and keep the remaining amount to themselves. Interestingly, Vinkers et al. (2013) also varied the timing of the social decision making tasks, where half of the male participants worked on the tasks immediately after the TSST-G/Placebo-TSST-G (in order to examine rapid non-genomic effects of cortisol) and the other half worked on the tasks 75 min after the cessation of the TSST (in order to examine slow genomic effects of cortisol; see Joels and Baram, 2009 for a description of the different phases of the stress response). They found that the effects of threat on behavior in the ultimatum game were time-dependent. In the direct aftermath of the threat—and in line with the findings of von Dawans et al. (2012)—no effects of threat were found. By contrast, in the delayed condition, threatened participants rejected fewer unfair offers (i.e., less altruistic punishment). In the Dictator Game, a time-independent negative effect of stress on prosocial behavior was found: Stressed participants donated less money to a charitable organization.

A different type of prosocial behavior was investigated in a vignette study by Starcke et al. (2011). This study examined whether social-evaluative threat affects prosocial behavior in everyday moral decision making. Participants had to decide on everyday moral dilemmas, each offering a more egoistic and a more altruistic decision alternative (i.e., “You find a 20$ note on the pavement. Then you see a homeless man looking for food in the dustbin. Would you give him the money?”; Starcke et al., 2011, 217). The results revealed no significant differences between the stressed TSST and the not-stressed control group. There was, however, a significant negative correlation between cortisol and the morality of the decisions, indicating that participants (male and females) with a stronger cortisol reaction made more egoistic decisions in the dilemmas.

Taken together, the three TSST-studies described above suggest that social-evaluative threat can influence prosocial behavior in several different ways. The direction of this effect seems to depend on specific conditions. Thus, social-evaluative threat has been found to lead to more prosocial decisions when the decision is targeted toward single individuals (von Dawans et al., 2012), but not when it is targeted toward a charitable organization (Vinkers et al., 2013) or involves just predicting one’s own behavior in hypothetical situations (Starcke et al., 2011). Notwithstanding the importance of these studies, it has to be noted that they all examined prosocial behavior in a rather constrained fashion, that is, by using vignettes (Starcke et al., 2011) or decision paradigms from behavioral economics (von Dawans et al., 2012; Vinkers et al., 2013). By contrast, numerous studies from social psychology have examined spontaneous prosocial behavior in a more unconstrained setting. For example, in a prototypical study, the experimenter accidentally spills some pencils on the floor, and the dependent variable is whether participants help him or her to pick them up (e.g., Greitemeyer and Osswald, 2010). Hence, it would be an interesting avenue for future research to study the impact of social-evaluative threat on spontaneous prosocial behavior using paradigms from social psychology. Also, this research might examine whether the effects of social-evaluative threat on prosocial behavior are mediated by the accessibility of prosocial thoughts.

## DISCUSSION

The present review aimed at evaluating the potential of the TSST to study the interplay between social and neurophysiological factors during threatening social interactions. To this end, we reviewed research using the TSST to examine either the effects of social factors on threat-related neurophysiological stress responses, or the effects of social-evaluative threat and neurophysiological stress responses on social processes.

### SOCIAL-EVALUATIVE THREAT—INSIGHTS FROM RESEARCH EMPLOYING THE TSST

The most robust finding in all of the reviewed studies is that social-evaluative threat leads to a strong neuroendocrine stress reaction as indicated by elevated levels of cortisol and high levels of self-reported stress. This is remarkable since in all of these studies the participation in the TSST, of course, had no real life consequences for the participants. Thus, none of the participants would have been excluded from a social group or would have lost his or her job if he or she failed in the TSST. Although no direct real life consequences emerge, it might be argued that at least to some extent the experienced stress in the TSST may stem from the anticipation of real life consequences in the future. Thus, participants may fear a similarly poor performance in a comparable real life situation (e.g., a job interview).

Luckily, however, studies employing the TSST have also examined which social factors can buffer neuroendocrine stress reactions. Most prominently, several studies have tested whether receiving social support works as a stress buffer. Somewhat counterintuitively, social support per se has been found to be often insufficient in buffering the neuroendocrine stress reaction, and the studies reviewed in this article have identified some important moderators like, for example, the relationship between support provider and recipient (e.g., Frisch et al., 2014), the type of support offered (e.g., Ditzen et al., 2007) or the sex of the support recipient (e.g., Kirschbaum et al., 1995). Apart from direct social support also other—more subtle—social processes, like social status (e.g., Gruenewald et al., 2006) or racial biases (e.g., Mendes et al., 2007) have been found to affect the neuroendocrine response or recovery. Interestingly, there is also preliminary evidence for contagion effects regarding the neuroendocrine stress response, that is, the participants’ neuroendocrine responses to stress resonate with the TSST committee members or with passive observers (Buchanan et al., 2012; Engert et al., 2014).

Social variables and processes do not only influence the neurophysiological reaction to threat, but social-evaluative threat and the corresponding neurophysiological responses can also backfire on social factors. Ironically, social-evaluative threat seems to have negative effects on the very social abilities that are particularly helpful to cope with that threat, like the recognition of emotions of others (Smeets et al., 2009; Tomova et al., 2014) or the anticipation of their behavior in strategic interactions (Leder et al., 2013). On the other hand, exposure to social-evaluative threat can also lead to functional protective responses, such as an increase in prosocial behavior (von Dawans et al., 2012).

Although the studies reviewed above substantially advance our understanding of social-evaluative threat, they also raise some conceptual issues that should be addressed by future research. In particular, these conceptual issues comprise (a) the dissociation of neurophysiological and psychological stress responses as well as (b) the specific mechanisms underlying the relationship between social variables and threat-related neurophysiological processes.

#### Dissociation of neurophysiological and psychological stress responses

The TSST reliably triggers increases in both neurophysiological and psychological stress indicators (e.g., Kudielka et al., 2007). However, especially in studies examining the stress attenuating influence of social support on stress reactions, a dissociation of both response levels has been found. In most of these studies (Kirschbaum et al., 1995; Ditzen et al., 2007; Taylor et al., 2010; Häusser et al., 2012; Frisch et al., 2014) social support effectively buffered the neurophysiological stress response but did not affect the self-reported stress levels—which always remained high. In line with this, TSST studies often do not find significant correlations between both indicators of stress. Eight studies reviewed in this paper directly tested the association between the neurophysiological and the psychological stress response. Only two of them (Gruenewald et al., 2006; Ditzen et al., 2008) report a significant correlation whereas in the other six studies cortisol was not related to self-reported stress (Kirschbaum et al., 1995; Roelofs et al., 2005; Ditzen et al., 2007; von Dawans et al., 2012; Frisch et al., 2014; Tomova et al., 2014). This absence of a direct relationship is confirmed in a review article by Campbell and Ehlert (2012) who found that only 27% of studies employing the TSST or similar stress induction paradigms have found a significant correlation between cortisol levels and self-reported stress.

In light of this dissociation, two questions arise: (1) Why do the neurophysiological and the psychological stress responses so rarely correlate? (2) And more specifically, why does social support buffer the neurophysiological stress response but is less likely to have an effect on the psychological stress response?

Regarding the first question, various reasons for the absence of a direct relationship between the neurophysiological and the psychological response to social-evaluative-threat have been discussed. For example, Hellhammer et al. (2009) argue that in addition to the involvement of brain structures related to the experience of emotions, many other neuroendocrine factors exert their influence on the stress-induced cortisol response. Hence, a low association of physiological and psychological stress responses is not very surprising. Further explanations aim at methodological issues as the timing of the assessment of self-reported stress (Hellhammer and Schubert, 2012) and some authors even doubt the link between cortisol and self-reported stress (e.g., Gruenewald et al., 2004).

Regarding the second question, the failure of social support to buffer the self-reported stress response might be—at least partially—explained by demand characteristics and self-reported biases. For example, since the aim of the TSST—stressing the participants—is quite obvious, participants may think that it is expected of them to report high levels of stress (e.g., Orne, 1962). But the opposite may also be true, due to self-protective mechanisms or avoidance motivations (e.g., Gramzow et al., 2003), participants may deny the level of stress experienced and indicate low amounts of stress. In both cases—either reporting more or less stress than actually experienced—an underestimation of the correlation between self-reported and physiological stress results and the effects of the experimental manipulation on the psychological level are undermined.

Moreover, a recent study by Het et al. (2012) suggests that high levels of cortisol in response to an acute stressor do not need to be associated with a negative emotional outcome, but may even have a mood-enhancing effect leading to less negative affect after the cessation of the TSST. Based on this finding, one could argue that participants who received no support prior or during the TSST indeed experienced higher levels of subjective stress than supported participants, but that these higher levels of subjective stress were buffered by the higher cortisol response. As a result, similar levels of subjective stress are reported by unsupported and supported participants. Although these explanations are speculative, they may serve as a starting point for further research. Furthermore, as the results of the study by Ditzen et al. (2008) suggest, moderators on the psychological level, like the attachment style, should also be considered. Particularly secure attached individuals might profit more from social support on the neuroendocrine as well as on the subjective level.

Beyond the potential reasons for the dissociation, the question remains as to whether social support can be claimed to be effective when it only buffers the neuroendocrine reaction but not the subjectively experienced stress response. Stated differently, what is the relative impact of (a) neurophysiological and (b) psychological stress on health and general well-being? Future research is needed to address this question. Finally, there is one interesting implication of the finding that social support is frequently ineffective in buffering the subjectively experienced stress response: People may fail to capitalize on social support because in their experience it does not make them feel better—thereby giving away the benefits on the neurophysiological level (Frisch et al., 2014).

#### Underlying mechanisms

Another important limitation of the reviewed studies is that most of them do not address the specific mechanisms underlying the relationship between social variables and threat-related neurophysiological processes.

Regarding the effects of social variables on neurophysiological processes, it might be worthwhile to study, for example, by which pathways social support and social identification affect the neurophysiological response. One promising key to understand why social support can be effective would lie in examining its role in situational appraisal processes (e.g., Ben-Zur and Michael, 2007; Gallagher et al., 2014).

Regarding the influence of threat and the corresponding neurophysiological response on social processes, the underlying mechanisms are also largely unknown. Although most studies do report a correlational relationship between cortisol and the social processes under study, only in one of the studies has a formal analysis of mediation been conducted (Leder et al., 2013) following the Baron and Kenny (1986) approach. But, of course, even this analysis is not suited to establish causality, since it is essentially correlational. In order to unequivocally establish causality, a valuable alternative to this measurement-of-mediation design is to experimentally investigate the proposed casual chain (Spencer et al., 2005). Note that, from a neurophysiological point of view, the effects of social-evaluative threat on social cognition and social behavior may be due to (a) an increase in cortisol levels (via the HPA axis), (b) an increase in noradrenergic activity (via the SAM axis), or (c) an interaction of concurrent glucocorticoid and noradrenergic activity. To disentangle these processes, researchers can pharmacologically manipulate both glucocorticoid activity and noradrenergic activity, for example, by the administration of hydrocortisone and yohimbine, thereby employing a 2 × 2 experimental design (e.g., Schwabe et al., 2012). Another method for disentangling the neurophysiological effects of glucocorticoid and noradrenergic activity is to selectively suppress either the glucocorticoid or the noradrenergic stress response. For example, the glucocorticoid stress response can be suppressed by employing the dexamethasone suppression test (e.g., Andrews et al., 2012), whereas noradrenergic activity can be suppressed by propranolol administration (Andrews and Pruessner, 2013). Thus, future studies might examine the neurophysiological underpinnings of social-evaluative threat by exposing participants to the TSST while simultaneously suppressing either the glucocorticoid or the noradrenergic stress response.

### EVALUATION OF THE TSST—INSIGHTS FROM RESEARCH ON THREAT IN SOCIAL INTERACTIONS

In this review, we sought to evaluate the potential of the TSST—which is the gold standard in biopsychological stress research (Kudielka et al., 2007)—as a method to examine the interplay between neurophysiological and social factors in threatening social interactions. From the studies reviewed above, we also can learn a lot about the TSST as an experimental paradigm, with respect to its range of applications, strengths and weaknesses. In what follows, we will summarize these insights and will evaluate the TSST. Let us start with the weaknesses of the TSST.

#### Weaknesses of the TSST

First, the scope of the TSST is limited to inducing a specific kind of threat, namely social-evaluative threat. If aiming at examining the consequences of, for example, threats to physical integrity, other paradigms might be more appropriate, such as the cold-pressor test in which participants immerse their hand for a few minutes into ice water (first described by Hines and Brown, 1936) or the socially evaluated cold-pressor test (Schwabe et al., 2008) which combines the cold-pressor test and TSST elements.

Second, a further problem of the TSST is that it actually contains two stress-inducing-elements: Apart from being socially evaluative, the TSST situation is highly uncontrollable for the participants. Thus, participants cannot influence their potential negative evaluation and only partially know what tasks they have to face and how things will proceed (Dickerson and Kemeny, 2004). Indeed, in many real life situations social-evaluative threat is tightly coupled with uncontrollability, since the behavior of the interaction partners in such situations can almost never be completely foreseen. However, uncontrollability and social-evaluative threat might still have independent effects, and these effects might differ with respect to different social processes. A way to remove or at least reduce the uncontrollability component could be to tell participants beforehand the exact procedure of the TSST (they could even been shown a video of the procedure).

Third, the TSST is not well suited for repeated measures designs: The HPA axis has been found to be highly sensitive to the effects of repeated stimulation with TSST and reacts with habituation to it (Pruessner et al., 1997; Schommer et al., 2003). This should be kept in mind when planning on using the TSST more than once in a sample.

Fourth, the findings of Buchanan et al. (2012) showing that the TSST can elicit an empathic stress reaction of the committee members—even at the neuroendocrine level—could be a problem for the internal validity of the TSST. Quite obviously, from a methodological point of view, the committee members’ behavior should be exactly identical for all participants. Yet, the findings of Buchanan et al. (2012) suggest that this might not be the case since the committee members tend to contagiously catch the stress of the participants. Whereas this contagion of stress across individuals is adaptive for coordinating the behavior of groups, it is problematic with regard to the internal validity of the TSST. However, these weaknesses of the TSST are balanced by several methodological strengths.

#### Strengths of the TSST

The TSST can be described as a reliable and effective, highly standardized psychosocial stress induction tool that simplifies the comparison and integration of findings of different studies but is still very flexible, so it can be used to study a variety of research questions.

First, the TSST leads reliably to a strong cortisol response as has been demonstrated in previous studies (Dickerson and Kemeny, 2004) as well as in all of the reviewed studies. Moreover, it is very effective, since about 70–80% of participants show a two- to threefold increase in cortisol levels (Kudielka et al., 2007). However, as we have seen in some of the studies (e.g., Smeets et al., 2009), it can be important to distinguish between high- and low-cortisol responders or to exclude cortisol non-responders (for discussion of exclusion criteria, see Miller et al., 2013). Hence, upon deciding to employ the TSST as a paradigm to induce social-evaluative threat, one can be sure that the basic precondition—the existence of a solid and high physiological and psychological stress reaction—is met for the majority of participants. Moreover, although it causes a strong physiological and psychological stress reaction, the TSST is still in accordance with established ethical research standards, like the declaration of Helsinki.

Second, the TSST protocol is highly standardized. The procedure is well-documented and since no special apparatuses (apart from a video camera and a microphone) or questionnaires are needed it can be easily applied in nearly every laboratory.

Third, this high degree of standardization allows for comparisons between different studies in one field of research. This facilitates the integration of these findings in reviews and meta-analysis as well as the replication and extension of previous studies.

Fourth, notwithstanding the high degree of standardization, the TSST is still flexible and can be adapted to the specific needs of the researcher, thereby paving the way to investigate a huge variety of research questions (e.g., examining the effectiveness of social support, or the existence of empathic stress reactions). Hence, to date several variations of the TSST exist for specific research questions (e.g., different control groups, group version) or populations of participants (e.g., children, older adults). One frequent employed variation is the TSST-G (von Dawans et al., 2011). For example, the TSST-G can be used to study the effects of social-evaluative threat in groups of participants (e.g., Häusser et al., 2012). Moreover, due the simple composition of four main elements (i.e., anticipation/preparation period, free speech task, mental arithmetic task and social-evaluative component) (Kirschbaum et al., 1993), the TSST can be adapted to the specific research question and context of each study by simply altering these elements or by adding new elements. For example, as we have seen in the research on the effectiveness of social support, it was possible to add a supportive element (i.e., friend, stranger or confederate) to the TSST protocol. Moreover, the relationship between the TSST committee and the participant or even between the participants in a TSST-G can be manipulated (Häusser et al., 2012; Frisch et al., 2014). Furthermore, the temporal sequence of the three phases of the TSST (i.e., preparation, speech task, math task) allows the measuring of behavior or self-reported emotions/cognitions at different time points and may help to reveal time-dependent effects: (a) Before the participants are told about the TSST (baseline), (b) after the preparation phase (anticipatory stress reaction), (c) during the two task (stress reaction) (d) directly and after the TSST (post-stress reaction) and (e) at several measurement points after the TSST (recovery reaction) (e.g., Kirschbaum et al., 1993; Hellhammer and Schubert, 2012).

Taken together, the TSST is perfectly suited for investigating the complex interplay of social-evaluative threat, social processes, and neurophysiological stress responses. We hope that our review article will stimulate new research directions and propel this field forward.

## Conflict of Interest Statement

The authors declare that the research was conducted in the absence of any commercial or financial relationships that could be construed as a potential conflict of interest.
